# Comparative Analyses of Sperm DNA Methylomes Among Three Commercial Pig Breeds Reveal Vital Hypomethylated Regions Associated With Spermatogenesis and Embryonic Development

**DOI:** 10.3389/fgene.2021.740036

**Published:** 2021-10-06

**Authors:** Siqian Chen, Shuli Liu, Siyuan Mi, Wenlong Li, Shengli Zhang, Xiangdong Ding, Ying Yu

**Affiliations:** Key Laboratory of Animal Genetics, Breeding and Reproduction, Ministry of Agriculture and National Engineering Laboratory for Animal Breeding, College of Animal Science and Technology, China Agricultural University, Beijing, China

**Keywords:** pig, sperm, DNA methylation, hypomethylated region, comparative epigenomics

## Abstract

Identifying epigenetic changes is essential for an in-depth understanding of phenotypic diversity and pigs as the human medical model for anatomizing complex diseases. Abnormal sperm DNA methylation can lead to male infertility, fetal development failure, and affect the phenotypic traits of offspring. However, the whole genome epigenome map in pig sperm is lacking to date. In this study, we profiled methylation levels of cytosine in three commercial pig breeds, Landrace, Duroc, and Large White using whole-genome bisulfite sequencing (WGBS). The results showed that the correlation of methylation levels between Landrace and Large White pigs was higher. We found that 1,040–1,666 breed-specific hypomethylated regions (HMRs) were associated with embryonic developmental and economically complex traits for each breed. By integrating reduced representation bisulfite sequencing (RRBS) public data of pig testis, 1743 conservated HMRs between sperm and testis were defined, which may play a role in spermatogenesis. In addition, we found that the DNA methylation patterns of human and pig sperm showed high similarity by integrating public data from WGBS and chromatin immunoprecipitation sequencing (ChIP-seq) in other mammals, such as human and mouse. We identified 2,733 conserved HMRs between human and pig involved in organ development and brain-related traits, such as *NLGN1* (neuroligin 1) containing a conserved-HMR between human and pig. Our results revealed the similarities and diversity of sperm methylation patterns among three commercial pig breeds and between human and pig. These findings are beneficial for elucidating the mechanism of male fertility, and the changes in commercial traits that undergo strong selection.

## Introduction

Pigs are an important source of fats, proteins, and human biomedical models (Swindle et al., 2012). After a long artificial and natural selection period, considerable phenotypic differences have emerged between pig and human in morphology, physiological structure, and behavior. Identifying epigenetic markers subject to evolution in different pig breeds will contribute to elucidating the epigenetic mechanism of important economic trait changes and supporting pigs as human biomedical models.

DNA methylation is the most stable and commonly studied epigenetic marker (Curradi et al., 2002), which participates in genome imprinting, silencing of the transposon element, and transcription inhibition (Barlow, 1993; Zamudio et al., 2015). Previous studies have shown that DNA methylation plays an important role in the growth, fertility, and health of pigs (Wang et al., 2017). In addition, by comparing epigenome-wide skeletal muscle DNA methylation profiles in distinct metabolic types of pig breeds, Ponsuksili et al. (2019) demonstrated that breed-specific methylated genes are linked to muscle metabolism and trigger extensive compensatory processes.

Sperm is an important heritable lineage. The comparison of sperm DNA methylomes in the whole genome across different pig breeds is unknown. Thus, to fill this gap, we conducted WGBS of sperm DNA samples from different pig breeds to explore the potential genetic mechanisms of phenotypic diversity and male fertility. Duroc, Landrace, and Large White are three common commercial pig breeds known for their excellent production performance under long-term artificial selection. Comparing breed-specific epigenomic markers in three commercial pig breeds helps us to understand how epigenetic regulation leads to phenotypic changes during evolution.

**FIGURE 1:**
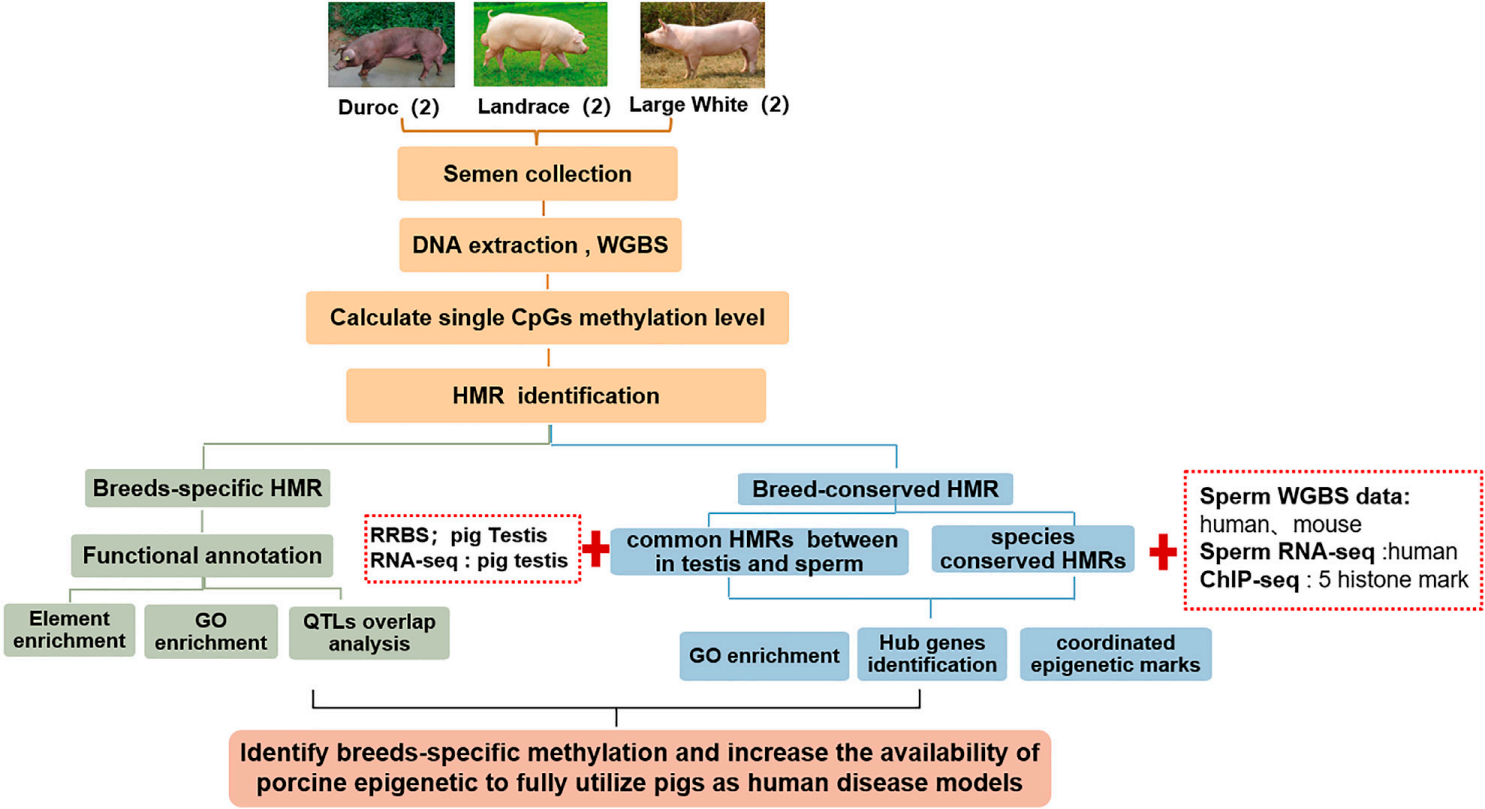
Schematic overview of this study. We identified breed-specific/breeds-conserved HMRs of pigs using whole-genome bisulfite sequencing of six sperm samples from three pig breeds in Landrace, Duroc, and Yorkshire. Thereafter, we annotated them by integrating GO/KEGG, traditional quantitative trait loci (QTL) and transcriptional factor binding sites. Finally, we analyzed changes of HMRs in mammalian evolution by integrating WGBS public data on humans and mice.

This study aimed to: 1. Investigate the sperm DNA methylomes using WGBS and analyze DNA methylation variation in three pig breeds—Landrace, Duroc, and Yorkshire; 2. Excavate breed-specific hypomethylated regions and identify genes that are enriched by lineage-specific hypomethylated regions around promoters; 3. Further identify the epigenetic biomarkers by integrating public reduced representation bisulfite sequencing (RRBS) data in pig testis; 4. Combine public data from WGBS and chromatin immunoprecipitation sequencing (ChIP-seq) in other mammals, the changes in sperm DNA methylation across mammalian evolution, and the epigenetic mechanism of pigs as human medical models (Figure 1).

## Materials and Methods

### Sample Collection and Sequence Library Preparation

Six sperm samples were collected from three pig breeds in Landrace, Duroc, and Large White (3 breeds × 2 individuals). The pigs were raised using the same feed type on the same farm. Semen samples were collected by professional artificial insemination personnel according to a standardized procedure with artificial vaginas. Genomic DNA was extracted using a salt-fractionation protocol. DNA quality was assessed using a 2,100 Bioanalyzer (Agilent Technologies, Santa Clara, CA, United States) and a spectrophotometer (NanoDrop Technologies, Rockland, DE). The qualified genomic DNA were spiked with unmethylated lambda DNA and fragmented into 200–300 bp, followed by terminal repair, the addition of 3′ A and adapter ligation. The DNA fragments were treated twice with bisulfite using an EZ DNA Methylation-Gold™ kit (Zymo Research, Irvine, CA, United States), under the manufacturer’s instructions. Then the DNA fragments were amplified by PCR to screen the qualified library and sequenced using a paired-end 150 bp flow cell on an Illumina HiSeq X Ten machine (PE-150bp FC; Illumina, San Diego, CA, United States).

### WGBS Data Processing and Hypomethylated Region Identification

The public WGBS data used in this study included human sperm (GSE30340 and GSE57097) (Hammoud et al., 2014; Molaro et al., 2011) and mouse sperm (GSE49623) (Molaro et al., 2011). Three RRBS data of Landrace testis tissues from the NCBI GEO database (GSE129385) (Wang and Kadarmideen, 2019). FastQC v 0.11.2 (https://www.bioinformatics.babraham.ac.uk/projects/fastqc/) and Trim Galore v 0.4.0 (https://www.bioinformatics. babraham. ac. uk/projects/trim _galore/) was used to assess the quality of the sequence data and filter low-quality reads (*q* < 30), respectively. The cleaned data were then mapped to the respective reference genomes: sscrofa11.1 (pig), hg38 (human), and mm10 (mouse), using bowtie2 under the Bismark software (0.14.5) with default parameters (Langmead et al., 2009; Krueger and Andrews, 2011). Furthermore, bismark_methylation_extractor was employed to obtain methyl CpG site information.

CpG sites with coverage greater than five were used for the HMR analysis. We used tools from the MethPipe package to identify HMRs, as described previously (Song et al., 2013). HMRs were inferred according to the methylation level and coverage at individual cytosines, using the HMM algorithm, which is trained using the Baum-Welch algorithm and posterior decoding (Rabiner, 1989). We only kept HMRs with an average regional methylation level of less than 20% and at least five CpG sites for further analysis.

### Identification of Conserved/Specific HMRs

The conservation and variability of HMR was analyzed using BEDTools (version 2.26.0) (Quinlan and Hall, 2010). This study required conserved HMRs overlapping more than 25% of the base pair unless otherwise stated. Specific and conserved HMRs were identified according to the number of overlapping HMRs across breeds, tissues, or species.

### RNA-Seq Data and Gene Expression Quantification

The transcriptomes of pig testes (PRJEB33381) and human sperm (PRJNA573604) were collected from a public database. Thereafter, the RNA-seq reads were mapped to the respective reference genomes of pigs (sscrofa11.1) and human (Hg38) using the HISAT2 software (Kim et al., 2015). Finally, we obtained FPKM using StringTie software to quantify gene expression levels for downstream analyses (Pertea et al., 2015).

### DNA Motif Enrichment

DNA motif enrichment analysis of breed-specific HMRs was conducted in three pig breeds. Moreover, we identified overrepresented DNA sequences using MEME suite 5.0.5 with the following parameters:’ -dna -minw 8 -maxw 20 -mod zoops -objfun de -nmotifs 10’ (Bailey et al., 2009). In addition, 2000 HMRs were randomly selected as the control region. We compared motifs against known motifs using the Tomtom of MEME suit software to identify enriched motifs. The databases of known motifs consist of jolma2013 motifs (Jolma et al., 2013), HOCOMOCOv11 full HUMAN mono meme format motifs (Kulakovskiy et al., 2018) and JASPAR2018 CORE vertebrate non-redundant motifs (Khan et al., 2018). Significant motifs were identified at *p* < 0.05.

### ChIP-Seq Data and Epigenetic Features Analysis

Histone data were downloaded from the SRA dataset, including five histone modifications: H3K27me3, H3K4me1, H3K4me3, H3K36me3, and H3K27ac (PRJNA173071 and PRJNA281061). First, we obtained clean reads using the Trim Galore software. The clean reads were mapped to hg38 (human) using the Bowtie2 software. Then, the Picard tool (http://broadinstitute.github.io/picard/) was used to remove duplicated reads with parameters ‘REMOVE_DUPLICATES = true’. Histone peaks were obtained using the MACS2 software. The overlap between the HMR datasets and five histone modifications was identified using BEDTools, as previously described.

### Other Downstream Bioinformatics Analysis

The proportion of breed-specific HMRs that fell completely within the QTLs were calculated. The QTLs were downloaded from the Pig QTL database (https://www.animalgenome.org/cgi-bin/QTLdb/SS/index). Genes with promoter HMRs were annotated using the online software DAVID (http://metascape.org/). Significant GO terms and pathways were identified based on *p* < 0.05. GO terms were visualized using the R package GOplot (version 1.0.2) (Walter et al., 2015). Thereafter, the protein-protein interaction network (PPI) encoded by important genes was investigated using the STRING Genomics database (https://www.string-db.org/) and identified hub genes with a higher degree of connectivity using CytoHubba under the Cytoscape software (Shannon et al., 2003).

## Results

### General Characteristics of Sperm DNA Methylome

In this study, we conducted WGBS of individual sperm DNA samples from three commercial pig breeds—Landrace, Duroc, and Large White. We obtained 204 to 307 million unique mapped reads with an average coverage from 12.21 to 18.43× (Supplementary Table S1). An overall methylation level of 86.20–87.80% was observed for all CpG sites in the three pig breeds, and non-CpG methylation levels (CHG and CHH) of Landrace (5.90%) were higher than those of Duroc (1.40%) and Large White (1.55%). Moreover, the bisulfite conversion rates of all samples were greater than 99%; therefore, we faithfully captured the patterns of porcine sperm genomic DNA methylation.

**FIGURE 2:**
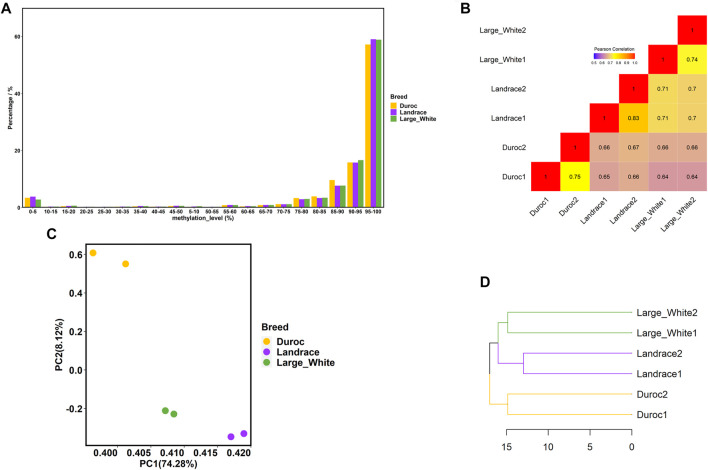
Porcine sperm DNA methylome characteristics. **(A)** Distribution of methylation levels of CpG sites (only CpG sites covered by at least five reads were included). **(B)** Correlation analysis between each sample using common CpG sites. **(C)** PCA of methylation levels in six samples using common CpG sites. **(D)** Hierarchical clustering of three pig breeds based on the methylation level of promoter regions.

In general, we observed that approximately 86% of the analyzed CpG sites were extremely hypermethylated (methylation level ≥80%) for all samples (Figure 2A), which was consistent with the pattern of sperm methylation in other mammals (Molaro et al., 2011; Song et al., 2013; Fang et al., 2019; Liu et al., 2019). Then, we compared the global methylation levels between pairs of samples at the common CpG sites within and among breeds, with a minimum coverage of five sequencing reads. As expected, DNA methylation varied more across breeds than within breeds (Figure 2B). The correlations were higher within breeds, ranging from 0.74 to 0.83. The correlations of DNA methylation among the three pig breeds were lower, with the correlations between Duroc and Large White being the lowest (*r* = 0.64). Principal component analysis (PCA) also demonstrated this phenomenon (Figure 2C). PC1 successfully divided samples into three clusters (Landrace, Duroc, and Large White) according to the variations among the three breeds, which explained 74.28% of the variance.

To study the evolution of DNA methylation among the three porcine breeds, we further investigated the promoter methylation levels of different breeds. A promoter region was defined as the segment 1.5 kb upstream and 0.5 kb downstream of transcription start sites (TSSs) (de Vooght et al., 2009; Shen et al., 2012; Zhou et al., 2021). The hierarchy in the sperm DNA methylation cluster (Figure 2D) was consistent with the known genetic relationship between the three porcine breeds (Cai et al., 2020). Figure 2D shows that, compared with Duroc, the similarity between Large White and Landrace was higher.

### Inter-Breed Variation and Conservation in Sperm DNA Methylome

**FIGURE 3:**
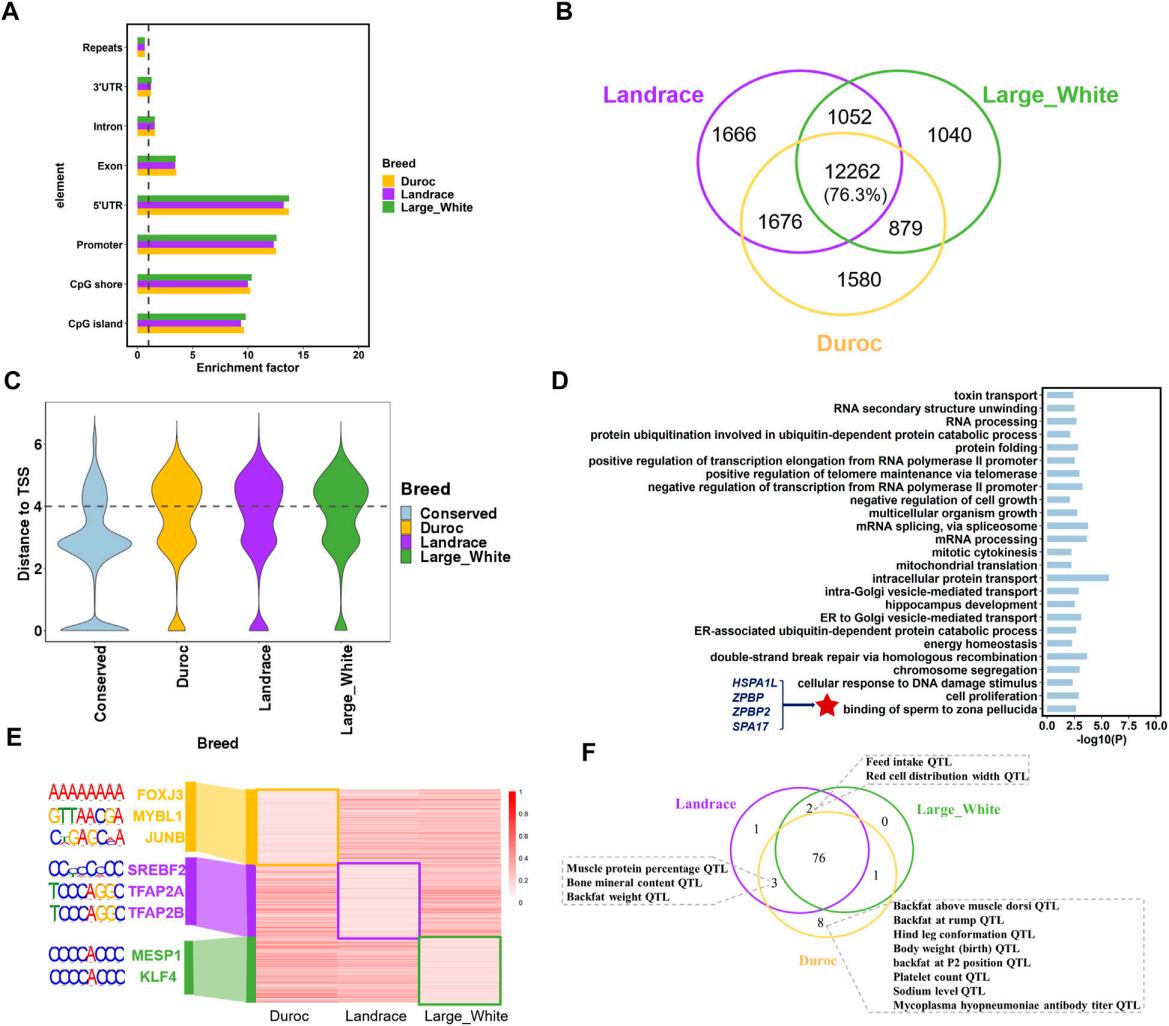
Porcine sperm hypomethylated regions (HMRs) characteristics. **(A)** The enrichment of HMRs across genomic elements. **(B)** The number of conserved HMRs of the three porcine breeds. **(C)** The distribution of distances from the different types of HMRs to closet TSS. **(D)** Go term (*p* < 0.05) analysis of genes associated with breeds-conserved hypomethylated (<20%) promoters (hypo-genes) in pigs. **(E)** The methylation level and the significant DNA motifs of breed-specific HMRs. **(F)** The overlapped pig QTLs and breed-specific HMRs in the three porcine breeds.

Hypomethylated regions (HMRs) are associated with gene activation and coincided with gene regulatory elements, including gene promoters and enhancers (Jones, 2012; Wagner et al., 2014); therefore, we identified HMRs (methylation level ≤20%) using the 2-state hidden Markov model with beta-binomial emission distributions as previously described (Song et al., 2013). The algorithm detected 15,233 to 16,656 HMRs with an average size range of 1,109–1,146 bp. Although HMRs were distributed throughout the genome, they were significantly enriched in the 5’ UTR, promoters, CpG islands, and CpG shores (*p* < 0.001; 1,000 times of permutation test) (Figure 3A). The distribution of HMRs in these genomic regions further indicated that HMRs play an important role in regulating transcriptional initiation and gene expression.

To analyze the difference in DNA methylation across pig breeds, we defined two categories of regions from HMRs: 1) breeds-conserved HMRs, containing a region that is hypomethylated in all breeds, and 2) breed-specific HMRs, which are regions that are hypomethylated in one breed compared to the other two breeds. As expected, most HMRs were conserved in the three porcine breeds, with an average of 76.3% in each breed (Figure 3B). Moreover, the genomic distribution of breeds-conserved HMRs and breed-specific HMRs was obviously different. A total of 49.62% of breed-specific HMRs were located in the distal regions (more than 10 kb away from TSS), contrary to 22.08% of breeds-conserved HMRs (Figure 3C). These results were consistent with high conservation at promoters, as reported in previous studies (Qu et al., 2018; Villar et al., 2015). In addition, this result also indicates that we need to pay attention to the HMRs located at the distal genome in future studies of breeds divergence. These breeds-specific regions may be located at distal regulatory element regions, thus affecting gene expression differences in breeds. Functional enrichment analysis revealed that the genes (*n* = 1800) with breeds-conserved HMRs in the promoters were engaged in basic cell function and fertilization, including energy homeostasis, cell proliferation, multicellular organism growth, and binding of sperm to the zona pellucida (Figure 3D, Supplementary Table S2). In the binding of sperm to the zona pellucida GO term, we found several genes associated with spermatogenesis and fertilization, namely, *HSPA1L* (heat shock protein family A member 1 like), *SPA17* (sperm autoantigenic protein 17), *ZPBP* (zona pellucida binding protein), and *ZPBP2* (zona pellucida binding protein *2*). *HSPA1L* has also been shown to be related to spermiogenesis in cattle, human, and mouse (Liu et al., 2019; Wang et al., 2020). This implies that DNA methylation patterns of key genes associated with spermatogenesis are highly conserved across species (Figure 3D, Supplementary Table S2).

We identified 1,580 Duroc-specific, 1,666 Landrace-specific, and 1,040 Large White-specific HMRs (Figure 3B). The genes with breed-specific hypomethylation in the gene promoter revealed a strong enrichment for transcription and development (Supplementary Table S3), such as post-embryonic development, kidney development, and regulation of transcription from RNA polymerase II promoter. Genes were also enriched in GO terms related to complex traits, such as high-density lipoprotein particle remodeling and prostate gland growth. Moreover, we discovered significant DNA motifs in breed-specific HMRs (Figure 3E, Supplementary Table S4). Landrace and large white HMRs were enriched for embryonic development and cholesterol synthesis. In the Duroc-specific HMRs, we identified significant motifs associated with skeletal muscle regeneration and male meiosis, such as *FOXJ3* and *MYBL1*.

To further investigate the relationship between breed-specific HMRs and complex traits, we examined the QTL regions of five categories (meat and carcass, health, exterior, production, and reproduction) of traits (*n* = 691) from the Pig QTL database (https://www.animalgenome.org/cgi-bin/QTLdb/SS/index). We observed that Duroc-specific HMR had higher overlapping QTL signals of production traits, meat, carcass traits such as body weight (birth) QTL, and backfat above muscle dorsi QTL. In addition, Duroc and Landrace had specific HMRs overlapping with muscle protein percentage QTL and backfat weight QTL (Figure 3F). These results were consistent with their varietal characteristics.

### Comparison of HMRs Between Porcine Sperm and Testis

**FIGURE 4:**
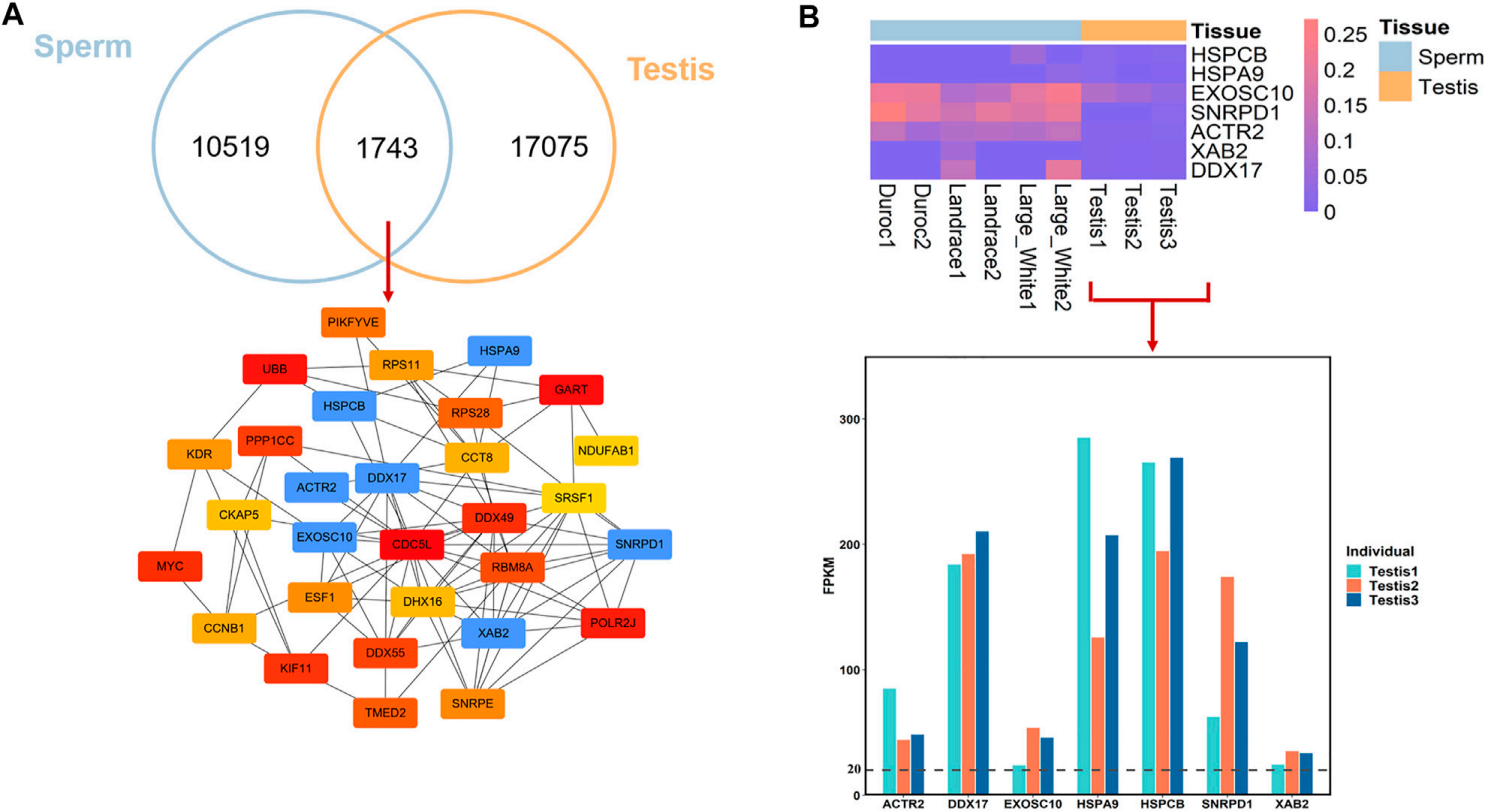
DNA methylation conservation and divergences between sperm and testis in pig. **(A)** Overlap of HMRs and hub genes identification. **(B)** DNA methylation level in promoter regions between sperm and testis and gene expression of hub genes in testis.

Testis is the organ which produces sperm, the male reproductive cell, and androgens, the male hormones (Schumacher, 2012). Moreover, spermatogenic activity co-varies with testis mass and sperm quality across (Pintus et al., 2015). To better understand the epigenetic mechanism that regulates male fertility and semen quality, we investigated the conservation and divergence of breeds-conserved HMRs in sperm and HMRs in Landrace testis. We found that 14.23% of HMRs (*n* = 1743) in sperm were common to the testis (Figure 4A). This result may imply that HMRs have higher tissue specificity. Functional annotation revealed that for genes with conserved HMRs at the promoters between sperm and testis, GO terms of mRNA splicing, cell proliferation, regulation of double-strand break repair via homologous recombination, and regulation of cell cycle during spermatogenesis (Supplementary Table S5).

To further investigate the important genes associated with the spermatogenesis process, we conducted PPI analyses and identified hub genes from genes with conserved HMRs at the promoter between sperm and testis. We found that among 1743 conserved HMRs, 30 genes with a higher degree of connectivity were hub genes (lower panel, Figure 4A). Several genes with hypomethylated promoter regions were highly expressed in the testis (Figure 4B). In particular, *EXOSC10* (exosome component 10) and *HSPA9* (heat shock protein family member 9) have been suggested to play important roles in male germ cell development (Jamin et al., 2017; Jeong et al., 2017).

### The Conservation of Breeds-Conserved HMRs Across Species

**FIGURE 5:**
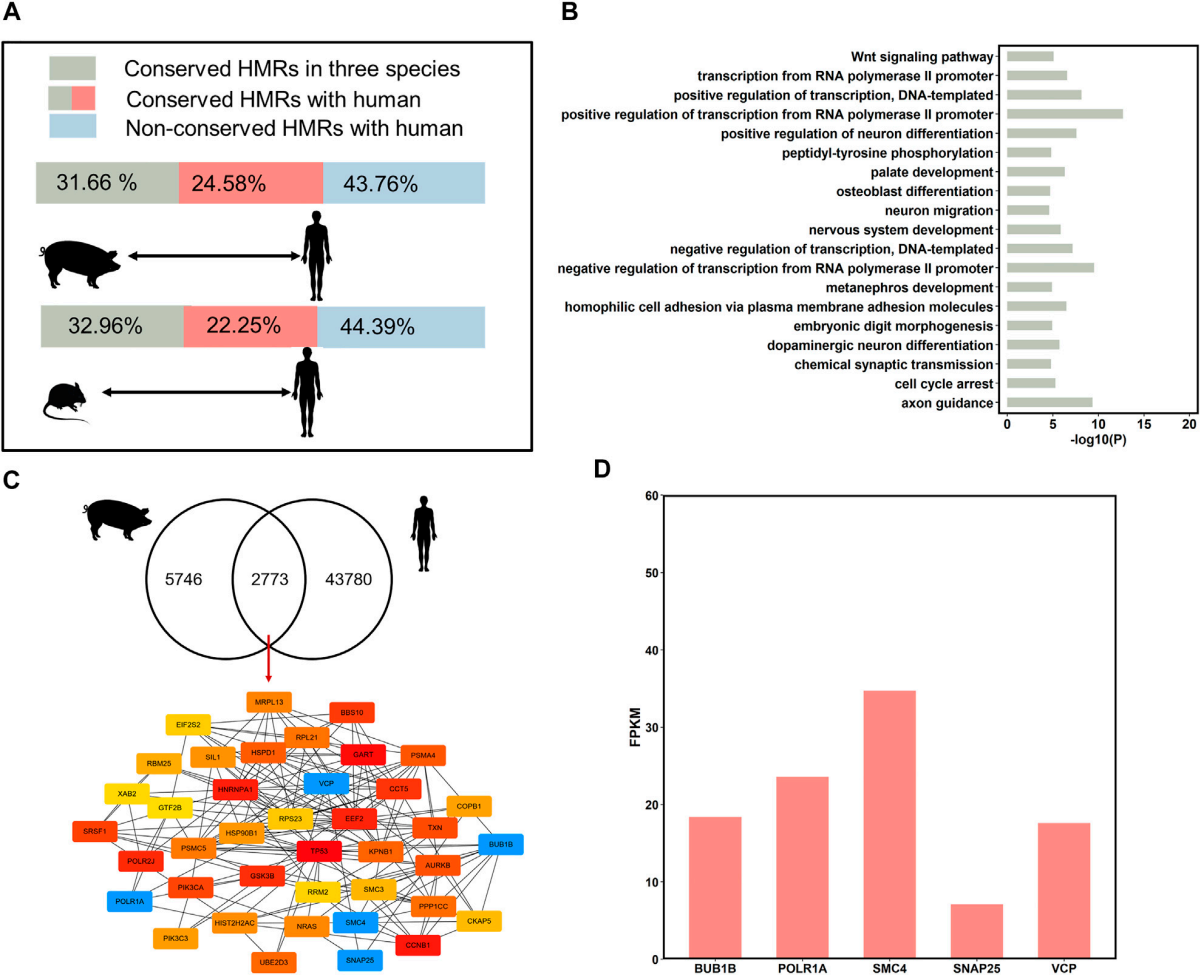
DNA methylation conservation and divergences of species. **(A)** Comparison HMRs in human to pig, and mouse. The green bars correspond to the conservative HMR percentage of three species in the pig and mouse. The green bars plus red bars correspond to the percentage of conserved HMRs in pig and mouse, compared to the human. The blue bars correspond to non-conservative HMR percentage in the pig and mouse, compared to the human. **(B)** Gene Ontology analysis of genes associated with conserved hypomethylated (<20%) promoters in three species (top 20, *p* < 0.05). **(C)** The hub genes with conserved HMRs between humans and the three pigs at the promoters (top 40). **(D)** The expression level of the important hub genes in human sperm.

HMRs around promoters can reflect the evolution of the mammalian epigenome (Qu et al., 2018); therefore, we compared HMRs in the sperms of three species, *i.e.,* pig, human and mouse, to explore the evolution of germline DNA methylation and epigenetic mechanisms underlying remarkable changes. Consequently, our results suggested that the proportion of conserved HMRs in three species only constituted 31.66–32.96% (Figure 5A), implying that the epigenome exhibits evolutionary changes and strong lineage-specific aspects across millions of years of evolution in the three mammals. Functional enrichment analysis showed that genes associated with three species-conserved HMRs in the promoters were enriched in transcription processing and embryonic development, such as transcription from RNA polymerase II promoter, nervous system development, and embryonic digit morphogenesis (Figure 5B, Supplementary Table S6). This suggests that genes involved in basic physiological functions and development processes tend to have conserved DNA methylation levels during mammalian evolution.

Pairwise comparison between human with pig and mouse revealed that an average of 61.14% (*n* = 8,553) of HMRs were conserved between humans and the other two species (Figure 5A). Interestingly, the percentage of conserved HMRs between pig and human (56.24%) was higher than that between mouse and human (55.61%); therefore, the use of pigs as an animal model for human diseases research is reasonable.

To investigate the similarities between human and pig sperm DNA methylation patterns and identify stable epigenetic markers, we compared the conservation of HMRs across the three pig breeds and humans. A total of 32.08% (*n* = 2,733) of the conserved HMRs across the three pig breeds were conserved in human (Figure 5C). Then, we obtained hub genes (top 40) from genes with conserved HMRs between pig and human at the promoters using Cytoscape (Figure 5C). We identified several important genes with hypomethylated regions at promoters highly expressed in human sperm (Figure 5D). These findings further suggest that these genes are related to mitosis progression and regulation of neurotransmitter release.

### Coordinated Epigenetic Marks at Conserved HMRs Between Human and Pig

**FIGURE 6:**
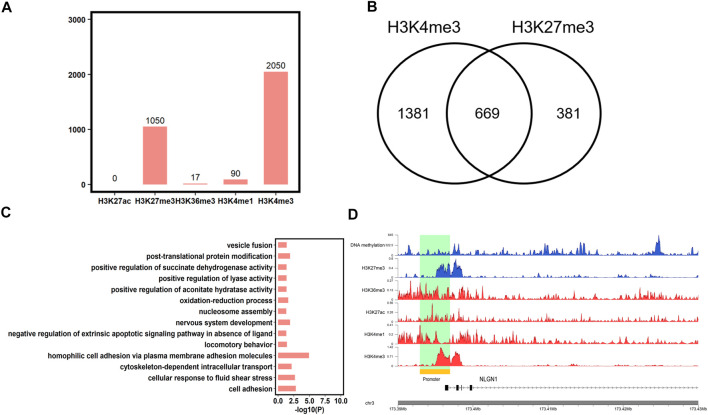
Epigenetic marks at conserved HMRs between human and pig. **(A)** The number of conserved HMRs between human and pig showed overlap with H3K4me1, H3K4me3, H3K36me3, H3K27ac, and H3K27me3. **(B)** The number of conserved HMRs simultaneously marked by H3K4me3 and H3K27me3. **(C)** Gene Ontology analysis of genes associated with conserved HMRs and bivalent domains at the promoters (*p* < 0.05). **(D)** Gene tracks showing DNA methylation, H3K4me1, H3K4me3, H3K36me3, H3K27ac, and H3K27me3 signal in human sperm at one representative poised gene, *NLGN1*.

In addition to DNA methylation, chromatin states also play an important role in spermiogenesis, fertilization, and development. Then, we analyzed the chromatin states of the conserved HMRs between human and pigs using five histone marks (H3K27me3, H3K4me1, H3K4me3, H3K36me3, and H3K27ac, downloaded from the SAR database). We found that 75.01% (*n* = 2050) of conserved HMRs between human and pigs overlapped with H3K4me3 marks, which is in accordance with the antagonistic relationship between DNA methylation and H3K4me3. In contrast, less conserved HMRs were enriched for H3K4me1 (*n* = 90) and H3K36me3 (*n* = 17) (Figure 6A). In addition, no conserved HMRs were enriched with H3K27ac (*n* = 0).

In addition, we found that some conserved HMRs (24.48%, *n* = 669) were simultaneously marked by H3K4me3 and H3K27me3 (Figure 6B), which are termed bivalent domains. Mounting evidence suggests that genes with bivalent domains are found in embryonic stem cells and germ cells and play an important role in initiating sexual differentiation, embryonic development, and completion of meiosis (Sin et al., 2015; Vastenhouw and Schier, 2012). We further found that genes containing HMRs and bivalent domains at promoters were significantly enriched in development and behavior terms, such as nervous system development and locomotory behavior (Figure 6C, Supplementary Table S7), as previously described (Vastenhouw and Schier, 2012). It implies that genes associated with organ specification are poised in germ cells, and that DNA methylation levels are highly conserved between species. Among them, *NLGN1* plays an important role in synaptic signal transmission and in neuropsychiatric disorders (Nakanishi et al., 2017). *NLGN1* was further found to have a conserved-HMR between human and pig, and contain a bivalent domain at the promoter in human sperm (Figure 6D). This result further suggested that *NLGN1* was regulated by the conserved epigenomic markers and may play an important role in brain-related traits between human and pig. Overall, These findings further support pig as an ideal animal model for human complex traits, particularly for brain-related diseases.

## Discussion

This study analyzed DNA methylation patterns in three commercial pig breeds (Durocs-Duroc, Landrace, and Large White) at a high resolution and investigated the relationship between pig and human with DNA methylation and histone modifications. In addition, the evolutionary properties of HMR in three commercial pig breeds, which may play an important role in embryonic developmental traits and adaptive traits, were analyzed. Our results also showed that most genes with conserved HMRs at promoters between human and pig are in the poised chromatin state (H3K4me3 and H3K27me3 bivalent), which are involved in brain-related traits.

Based on the comparison of sperm DNA methylome variation across three commercial pigs, our results showed that the divergence of sperm DNA methylomes fully recapitulates phylogenetic relationships, as previously reported for DNA sequences (Cai et al., 2020). Principal component analyses and hierarchical clustering demonstrated that breeds explain the largest amount of variation in sperm DNA methylomes, which PC1 could explained most (74.28%) of the variances. Taken together, these results suggested the importance of DNA methylation during breeds divergencies. Interestingly, we also noted that there were some differences in DNA methylation levels within breeds. Through comparing the intra-breed difference and conservatism of HMRs in three commercial pigs, we found that only the small proportion of HMRs are variable in intra-breed, which constituted 10.96–14.43% (Supplementary Figure S1). The methylation variations within breeds need to be analyzed through expanding the sample size for subsequent research.

In primate, the majority of species-specific DMRs locate outside promoters and have similar transcriptional potential as promoter DMRs (Mendizabal et al., 2016). In this study, we also found that many of the breed-specific HMRs were located in distal regions of genes. This was consistent with more rapid evolution of enhancers and slower changes at promoters in mammals (Villar et al., 2015). Moreover, we also observed that the important QTLs for carcass and production traits were obviously overlapped with breed-specific HMRs. For example, we found that 9 Duroc-specific HMRs were located with an important muscle protein percentage QTL for carcass and meat traits on chromosome 15 (BTA15, roughly located between 127.9 and 135.9 Mb) by comparing crossing porcine from the Pietrain and Duroc breeds (Choi et al., 2011). Six Duroc-specific HMRs were also located at the body weight (birth) QTL for production traits on chromosome 15 (BTA12, roughly located between 54.4 and 58.5 Mb) (Rothammer et al., 2014). These results imply that complex traits may be regulated by genetics and epigenetics, and breed-specific HMRs may be the candidate epigenetic markers that influence production and reproductive traits.

Through pig breeds-conserved HMRs in sperms compared with testis, we observed several conserved HMRs between pig sperm and testis close to or located in the promoter regions of important genes involved in spermatogenesis, such as *EXOSC10* and *HSPA9*. *EXOSC10* is a well-known target of autoantibodies in patients with systemic sclerosis (scleroderma) (Staals and Pruijn, 2010). Recent studies have also reported that *EXOSC10* is essential for normal growth-to-maturation transition in mouse oocytes and male germ cell proliferation and development (Jamin et al., 2017; Wu and Dean, 2020). This study also found that *EXOSC10* exhibited hypomethylated levels at the promoters of sperm and testis and high expression in the testis. Consistently, a previous study reported that the loss of *EXOSC10* in spermatogonia could lead to abnormal testicular development and a strongly decreased size (Jamin et al., 2017). *HSPA9* has also shown low methylation levels at the promoter and is expressed at a high level in the testis, which plays an important role in prophase I of spermatogenesis by binding to testis-specific *MAGEG2* (Melanoma Antigen Family G2) (Jeong et al., 2017). These results show that these conserved HMRs between the testis and sperm could be important candidate regions for the study of spermatogenesis and male infertility.

By analyzing the divergence of sperm DNA methylomes in three mammalian species: human, mouse and pig, we found that DNA methylomes were more highly conserved in pig and human than in human and mouse. Moreover, conserved HMRs between human and pig are also associated with GO terms related to development functions, particularly brain-related traits. This may be explained by brain-associated genes under strong selective constraints during the evolution of species (Cardoso-Moreira et al., 2019; Sarropoulos et al., 2019). For instance, SNAP25 (synaptosome-associated protein 25) is the core component of the soluble N-ethylmaleimide fusion protein attachment protein receptor (SNARE) and is associated with brain-related diseases, such as autism and schizophrenia (Braida et al., 2015; Ramos-Miguel et al., 2019; Washbourne et al., 2002). Moreover, we found that the conserved HMRs between human and pig were highly co-located with bivalent domains and were significantly enriched for GO terms of nervous system development and locomotor behavior. This was consistent with previous studies showing that developmental genes remain poised for later activation and are simultaneously marked by H3K4me3 and H3K27me3 in the male germline, such as the homeobox family of genes and the SRY-related HMG-box family of genes (Sin et al., 2015). In summary, these observations indicate that brain-associated genes may have highly conserved DNA methylation patterns across species evolution and are poised for activation at specific developmental stages.

## Conclusion

This study is the first to report a genome-wide comparative DNA methylation map of adult pig sperm in three commercial pig breeds using WGBS technology. Our results showed that sperm HMRs were highly conserved in the three commercial pig breeds. Moreover, our results indicated that breed-specific HMRs are related to phenotypic changes and economically complex traits for each breed. The conserved HMRs between pig sperm and testis close to or located in the promoter regions of important genes are mainly involved in DNA repair and spermatogenesis. Additionally, we found considerable similarities in DNA methylomes between pig and human sperms. The conserved HMRs between human and pig are related to brain-associated genes. In summary, our study of sperm methylomes in three commercial pig breeds contributes to understanding the mechanism of complex traits (particularly male fertility and brain-related traits) undergoing selection, further supporting pigs as human medical models from an epigenetic standpoint.

## Data Availability

The original contributions presented in the study are publicly available. This data can be found here: https://www.ncbi.nlm.nih.gov/geo/, GSE180099.
