# Supplementary figures and images for: Comparative Analyses of Sperm DNA Methylomes Among Three Commercial Pig Breeds Reveal Vital Hypomethylated Regions Associated With Spermatogenesis and Embryonic Development
Source: Front Genet. 2021 Oct 6;12:740036. doi: 10.3389/fgene.2021.740036 (PMC8527042; doi:10.3389/fgene.2021.740036)

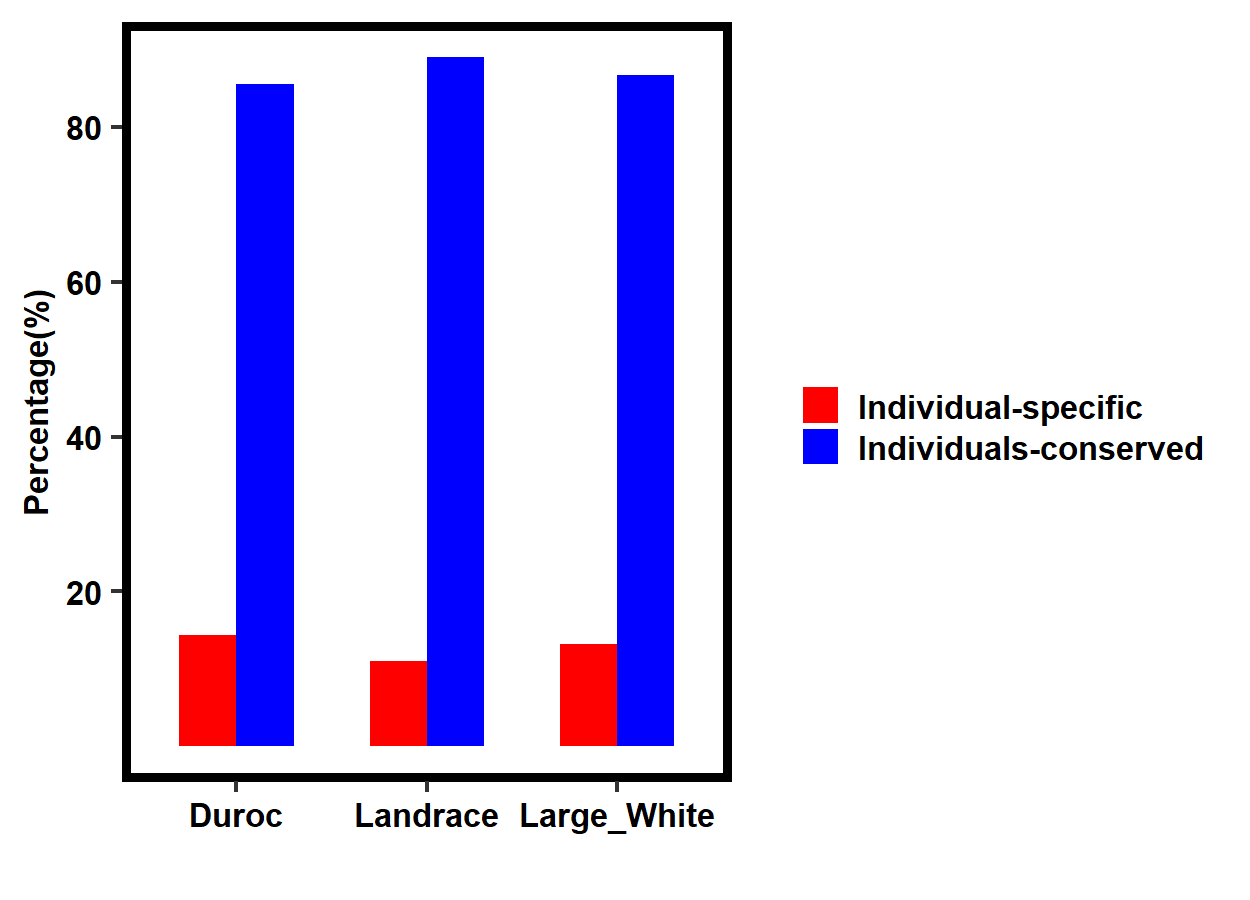

Supplement: Supplementary file 2 [file Image1.TIFF]
